# Genome Wide Identification and Comparative Analysis of the Serpin Gene Family in Brachypodium and Barley

**DOI:** 10.3390/plants9111439

**Published:** 2020-10-26

**Authors:** Shazia Rehman, Bodil Jørgensen, Ejaz Aziz, Riffat Batool, Samar Naseer, Søren K. Rasmussen

**Affiliations:** 1 Department of Botany, Rawalpindi Women University, 6th Road, Satellite Town, Rawalpindi 46200, Pakistan; 2 Department of Botany, Govt. Gordon College Rawalpindi, Rawalpindi 46000, Pakistan; 3 Department of Plant and Environmental Sciences, Faculty of Sciences, University of Copenhagen, 1871 Frederiksberg C, Denmark; boj@plen.ku.dk; 4 Department of Botany, Government Degree College Khanpur, Haripur 22650, Pakistan; ejaz.aziz.qau@gmail.com; 5 University Institute of Biochemistry and Biotechnology, PMAS, Arid Agriculture University, Rawalpindi, Rawalpindi 46300, Pakistan; riffatbatoolqau@gmail.com; 6 Department of Biology and Environmental Science, Faculty of Sciences, Allama Iqbal Open University, Islamabad 44000, Pakistan; samar_nasseer@yahoo.com

**Keywords:** serpin proteins, phylogenetic comparison, gene duplication, selection pressure, genome synteny

## Abstract

Serpins (serine protease inhibitors) constitute one of the largest and most widely distributed superfamilies of protease inhibitors and have been identified in nearly all organisms. To gain significant insights, a comprehensive in silico analysis of the serpin gene family was carried out in the model plant for temperate grasses *Brachypodium distachyon* and barley *Hordeum vulgare* using bioinformatic tools at the genome level for the first time. We identified a total of 27 BdSRPs and 25 HvSRP genes in *Brachypodium* and barley, respectively, showing an unexpectedly high gene number in these model plants. Gene structure, conserved motifs and phylogenetic comparisons of serpin genes supported the role of duplication events in the expansion and evolution of serpin gene family. Further, purifying selection pressure was found to be a main driving force in the evolution of serpin genes. Genome synteny analysis indicated that BdSRP genes were present in syntenic regions of barley, rice, sorghum and maize, suggesting that they evolved before the divergence of these species from common ancestor. The distinct expression pattern in specific tissues further suggested a specialization of functions during development and in plant defense. These results suggest that the LR serpins (serpins with Leu-Arg residues at P2–P1′) identified here can be utilized as candidates for exploitation in disease resistance, pest control and preventing stress-induced cell death. Additionally, serpins were identified that could lead to further research aimed at validating and functionally characterizing the role of potential serpin genes from other plants.

## 1. Introduction

The serpin superfamily is a member of the most ubiquitous and successful class of inhibitors and is found in all organisms, including animals, plants, bacteria, viruses and Archaea [1,2,3]. The majority of serpins inhibit serine proteases of the chymotrypsin family by employing a unique “suicide substrate” mechanism of irreversible inhibition [4,5], but few of them have evolved an ability to inhibit cysteine proteases as well [1,6]. Some other serpins have bifunctional activity or no inhibitory activity [7]. In general, serpins are large proteins (340–440 aa) with a molecular weight of 40–45 KDa, and they contain a flexible bait loop that can form covalent and irreversible complexes with proteases [1]. A typical inhibitory serpin consists of several α-helices and β-strands, together with an external reactive center loop (RCL). The RCL region is usually composed of 20–24 amino acid residues and is of critical importance in determining the inhibitory capacity of serpins [8]. Structural studies revealed that the P1–P1′ sessile bond in the RCL acts as bait for attacking proteases and the inhibitory specificity of serpins is mainly dependent on the identity of the active site residues (P4–P4′), in particularly P1 [1,4,9]. Thus, high diversity in these RCL residues may allow the serpins to target a wide range of proteases with different proteolytic specificities.

Several fundamental biological processes, including blood coagulating pathway and many other important proteolytic cascades in mammals, are controlled by serpins [10]. Serpin genes characterized so far from plants have inhibitory properties similar to that of animals, but target proteases for plant serpins have not been identified. In plants, serpins play an important role in defense against plant pathogens, having a great potential as breeding targets to improve disease resistance, as well as in food security, since they also appear vital to grain development. For instance, serpins identified in *Arabidopsis*, soybean and maize have been investigated for activity against the necrotrophic fungus *Botrytis cinerea* [11]. Similar defensive functions have been suggested against insects and pathogens for the serpins found in high concentrations in cereal grains and apple seeds [12,13,14]. In cereals, biotic stress-responsive serpins are likely to play an important role in disease resistance against *Fusarium culmorum*, which causes Fusarium head blight disease in barley [15]; *Magnaporthe oryzae*, which causes rice blast [16] and *Puccinia striiformis*, which causes stripe rust in wheat [17]. From pumpkin (*Cucurbita maxima*) phloem, Yoo et al. [18] isolated the CmPS-1 serpin, which has elastase-inhibiting activity, and they showed a negative correlation between increased levels of CmPS-1 and survival of the green peach aphid. Consistent with these results, an *Arabidopsis* serpin 1 (AtSerpin1) was shown to significantly reduce the growth of *Spodoptera littoralis* larvae and may be a good candidate for pest control [19]. A similar study has been carried out for three sorghum serpins (Sbser1, Sbser2 and Sbser3) showing activity against corn earworm [20]. These results suggest that plant serpins may inhibit the digestive protease activity of insects and the proteolytic enzymes of pathogens.

Based on previous studies on cereals, serpins are considered essential for grain development and quality. In barley, serpins act as storage proteins during grain filling and contribute up to 5% to the 7% lysine content of the total grain [1,21], and hence were suggested to be a target for breeding high-lysine barley. Wheat and rye grain serpins have evolved to inhibit proteases specifically adapted to the breakdown of grain prolamins [12]. Furthermore, serpins from barley and wheat grains have been assessed for inhibitory activity against chymotrypsin and cathepsin G. Thus, serpins found in grains participate in grain development by providing protection to storage proteins from digestion by insects and fungi [12,13].

Plant serpins are also found to participate in plant immunity as negative regulators of stress-induced cell death under biotic and abiotic stresses [22]. For example, AtSerpin1 was shown to act on Metacaspase 9 (AtMC9) in vitro and in vivo with the cysteine protease RD21 and to regulate stress-induced cell death in response to fungal attack [23,24,25]. More recently, AtSRP4 and AtSRP5 were identified as negative regulators of stress-induced cell death caused by bacteria [26]. Two other serpins, AtSRP2 and AtSRP3, were found to be associated with the regulation of growth responses in the presence of alkylating agents [27]. In another example, Bhattacharjee et al. [28] demonstrated that downregulation of the OsSRP-LR serpin (rice serpin1) shows exaggerated cell death upon exposure to pathogen infection, UV light and saline conditions. In line with this observation, Dhanushkodi et al. [29] reported increased papain-like cysteine protease activity, early nodule senescence and reduced plant growth with knockdown of the LR-type serpin (MtSer6) gene in *Medicago truncatula* under drought-stressed conditions. Hence, it was suggested that serpins constitute an important line of defense in plants under biotic and abiotic stresses.

The majority of plant serpins identified until now are known to be inhibitory and rarely perform non-inhibitory functions. In animals, the non-inhibitory serpins have diverse functions, including roles as hormone transporters [30], molecular chaperones [31] or tumor suppressors [32]. The presence of non-inhibitory serpins in plants may indicate their possible role in hormone regulation (as hormone transport molecules), protein storage or protein folding (as chaperones) [9]. In a recent study, Cohen and Fluhr [33] described the non-inhibitory function of a barley serpin Z4 for the first time and reported increased β-amylase activity due to interaction with serpin Z4 in response to heat and oxidative stresses. Both serpin Z4 and β-amylase are abundant seed proteins in many barley cultivars [34]. Thus, serpin Z4 also exhibits chaperone-like activity as well as an inhibitory function, demonstrating the dual biological role for cereal serpins [35,36]. A technical aspect of serpins is that protein Z is the dominant protein in beer foam, probably contributing to the foam stability and barley malt varieties with a high protein Z content can be selected for with the use of molecular markers [37].

Although serpin genes have been characterized from many plant species [38,39], a genome-wide comparison of the serpin gene family between *Brachypodium*, the model plant for temperate grasses [40], and barley, from which the first plant serpin was isolated and cloned [41], has never been performed. The aim of this study was to analyze the members of serpin gene family in *Brachypodium* and barley, based on genomic sequences and annotations. For this, we developed a nomenclature based on their chromosome location, identified gene duplication events and carried out genome synteny analysis and phylogenetic analysis of serpin genes. Additionally, expression patterns of serpin genes during development were evaluated based on publically available gene expression databases. Our analysis will demonstrate and expand our current knowledge about plant serpin genes for further functional inquiries and analysis.

## 2. Results and Discussion

### 2.1. Identification and Genomic Distribution of Serpin Genes

Based on the Plaza database (4.5) and Ensembl Plants database searches, a total of 27 serpin genes were identified from the *Brachypodium* genome and 25 serpin genes were found in barley. To maintain uniformity and avoid ambiguity, we proposed a new nomenclature in this study by numbering serpin genes according to their chromosomal locations (Table S1). In a previous study, plant serpins were named by using a five-letter abbreviation for species name (Brmdi for *Brachypodium distachyon*; Horvu for *Hordeum vulgare*) followed by the Z-numbering system ([1]; Table S2). The “Z” was used to denote sequence similarity with barley protein “Z” [34]. The Z-designation system for serpin nomenclature has been used in several previous reports [1,14,42,43]. In our study, the *Brachypodium* genome (ecotype Bd21) was found to contain 27 putative serpin genes, of which three appeared to encode non-inhibitory serpins and the remaining 24 were predicted to be inhibitory serpins (Table S1). Of these inhibitory serpins, 23 were classified as true serpins and one gene at locus Bradi4g15320 (BdSRP4-4) was shown to be a pseudogene with a short protein length of 80 amino acids. The serpins encoded by loci Bradi4g22020 (BdSRP4-7), Bradi5g16744 (BdSRP5-4) and Bradi5g16780 (BdSRP5-5) were predicted to be non-inhibitory due to differences in conserved residues in the reaction center loop (RCL).

In order to confirm the serpin gene copy numbers in *Brachypodium* (Bd21 reference genome), the ecotype Bd21-3 [44] was also included for comparison. In the present analysis, it was found that the Bd21-3 genome had 25 serpin genes, comprising 22 inhibitory serpins and three non-inhibitory serpins (Table S1). All serpins identified for Bd21-3 were homologous to the serpins of Bd21. Two out of 25 barley serpins (HvSRP3-3 and HvSRP4-5) were found to be non-inhibitory serpins, whereas 23 serpins were predicted to be true inhibitory serpins with a unique RCL sequence. These results are in accordance with previous findings of serpins in *Arabidopsis* and rice. In *Arabidopsis*, the gene At1g62170 (ArathZ5) was predicted to be a non-inhibitory serpin [1]. Similarly, rice has three non-inhibitory serpin genes (OsSRP-PLP, OsSRP-PTY and OsSRP-PGY) out of 14 serpins [9], whereas maize has a single non-inhibitory serpin gene (ZeamaZ9) [1]. Therefore, *Brachypodium* and barley are unusual by having twice the number of serpin genes compared to other plants. Moreover, *Brachypodium* was found to contain a single pseudogene of serpin at locus Bradi4g15320, which corresponds to the serpin pseudogenes in rice at loci Os01g16200 and Os11g11760. In the *Arabidopsis* genome, a substantial number of pseudogenes for serpins have been reported [1]. Pseudogenes are gene copies having genomic sequences similar to functional genes that have lost the capability to encode a functional protein. Such gene copies are usually generated by genomic duplication [1].

Previous in vitro analysis of inhibitory serpins from barley, wheat, oat, rye, pumpkin, apple and *Arabidopsis* showed that they can inhibit serine proteases of the chymotrypsin family [1,27]. In our analysis, the inhibitory serpin genes encoding serpins at locus Bradi1g14730 (BdSRP1-2), Bradi1g14740 (BdSRP1-3), HORVU3Hr1G104270 (HvSRP3-3), HORVU4Hr1G013480 (HvSRP4-1), HORVU4Hr1G013520 (HvSRP4-2), HORVU4Hr1G013550 ((HvSRP4-3) and HORVU4Hr1G013560 (HvSRP4-4) were very similar or identical in sequence to previously identified barley BSZX (Horvu ZX) [35] (Table S3), whereas HORVU5Hr1G111860 (HvSRP5-2) and HORVU5Hr1G111920 (HvSRP5-3) were quite similar to BSZ7/HorvuZ7, and we may point out their similar inhibitory properties. Moreover, the other inhibitory serpins of barley and *Brachypodium* at their corresponding loci were consistent with the results obtained for serpins in *Arabidopsis*, soybean, wheat and rice [1,9,12,36].

The inhibitory BdSRPs have diverse protein lengths, ranging from 313 amino acids (BdSRP4-3) to 540 amino acids (aa) (BdSRP4-13), with molecular weights (Mol.wt) of 34.99–57.76 kDa and isoelectric points (pI) varying from 5.09 to 10.04. On the other hand, the size of HvSRPs ranged from 181 (HvSRP7-2) to 520 (HvSRP1-1) amino acids and Mol.wt ranged between 20.03–55.29 kDa with pI values of 5.06–9.60. Details of all serpin genes, including gene IDs, locus position, size, Mol.wt and pI are shown in Table S1. Only a few showed a signal peptide indicating a cytoplasmic location of plant serpins. Most of plant serpins have molecular mass in the range between 39 and 43 kDa [1,45,46], which is in accordance with our present data. Overall, there was no difference between pI values of serpins in *Brachypodium* (5.19–10.04) and barley (5.06–9.60). These pI values complement the pI values observed for serpin genes in monocots (pI: 5.79) and eudicots (pI: 5.81) (reviewed by Roberts and Hejgaard, [1]). However, serpins with outlying values (with high pI) may indicate their possible functions relating to binding of negatively charged chemical species such as DNA [1]. Additionally, the markedly different pI values of serpin genes in our analysis suggest that specific serpins may be localized in specific compartments of the cell.

### 2.2. Chromosomal Distribution, Gene Structure and Conserved Motif Analysis

**Figure 1:**
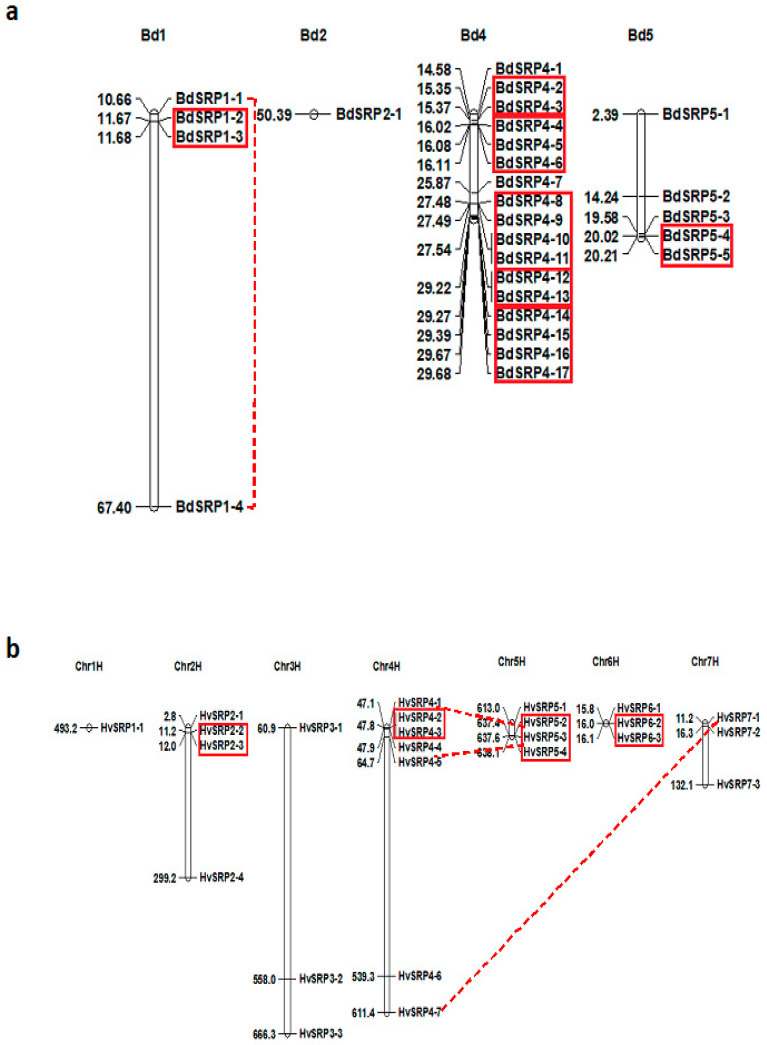
Chromosomal distribution of serpin genes in *Brachypodium* (Bd21) (**a**) and barley (**b**). Tandem duplicated genes are shown in boxes, and the segmentally duplicated genes are connected by dashed lines.

According to chromosomal distribution, a total of 27 serpin genes were identified from the entire *Brachypodium* genome (Table S1), which were found to be dispersed over four out of five chromosomes (Bd1, Bd2, Bd4 and Bd5) (Figure 1a). Among them, the highest density of serpin genes was found on chromosome 4, containing 17 BdSRP genes, whereas a single gene copy was recognized on chromosome 2. Chromosomes 1 and 5 had four and five serpin genes, respectively. In barley, a total of 25 serpin-like genes were mapped on seven chromosomes (Chr1H-Chr7H) (Figure 1b). Chromosomal location revealed that the serpin gene at locus HORVU1Hr1G071460 (HvSRP1-1) is located independently on chromosome 1H (Figure 1b). Chromosome 2H and chromosome 5H had four gene copies each, whereas three genes are localized on each of the 3H, 6H and 7H chromosomes. On the other hand, chromosome 4H had a maximum of seven serpin genes. Previous analysis of the *Arabidopsis* genome indicates six serpin genes which were distributed on three of the five chromosomes [1], whereas a majority of the 14 serpin genes identified from the rice genome are clustered on chromosome 11 [9].

The degree of amino acid sequence identity between BdSRPs ranged from 27% to 85% (Table S3), which is quite comparable with the percentage identity observed for rice serpins sequences (i.e., 24% to 87%) [9]. The serpin pairs with the highest similarity were BdSRP1-2/BdSRP1-3 (85%) and BdSRP4-10/BdSRP4-11 (82%). These serpin pairs were found to share maximum identity in their RCL sequence and represent neighboring genes on Chr1 and Chr4, respectively. Compared to *Brachypodium*, the identity of HvSRPs varied from 30% to 95% and highest level of identity was observed between the HvSRP4-7/HvSRP7-1 (95%) and HvSRP5-2/HvSRP5-3 (92%) pairs. This percentage sequence identity between serpin pairs in *Brachypodium* and barley also corresponds well with phylogenetic tree (discussed below), which further supports the suggestion that serpin genes arose via duplication during evolution in *Brachypodium* and barley. Comparison of the serpins between the two species HvSRP4-3 and BdSRP1-2 reveals that they share 93% and 84% RCL sequence identity with BSZX and hence are likely to have the same function.

**Figure 2:**
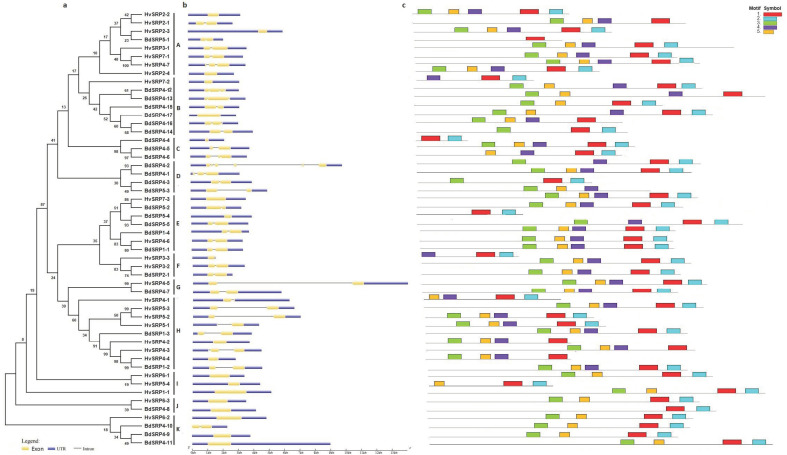
The phylogenetic relationships among serpin genes (**a**) and their exon/intron structures (**b**). The blue boxes represent the 5′-untranslated region (UTR) or the 3′-UTR, yellow boxes indicate exons and black lines exhibit introns. Conserved motifs in serpin proteins were identified using the MEME Suite program (**c**). The motifs are exhibited with specific colors. The tree was constructed with 1000 bootstrap replications using MEGA 6, based on the full-length protein sequence.

Based on the comprehensive analysis of gene structure, 13 out of 27 (48%) BdSRPs showed a single intron (Figure 2), consistent with the sequence analysis of barley serpin (HorvZ4/BSZ4), maize (Zeama9), Populus (PoptrZx) and rice (OsSRP-LRS) [1,9,47]. BdSRP4-2, encoded by locus Bradi4g14736, was the only gene with five introns, whereas the rest of them had two, three or no introns (Table S1). In the case of barley, no intron (14/25; 56%) was predicted in most of the HvSRPs, whereas the remaining serpin genes had either one or two introns (Table S1; Figure 2). Among rice serpin genes, OrysaZ12 at locus Os01g16200 was the only serpin without an intron [1]. Previous studies suggested that intronless genes have the ability to evolve rapidly through gene duplication events [48,49,50]. Moreover, serpin genes with a single intron represent the standard gene structure for plant serpins [1].

MEME Suite (v4.11.4) was used to discover the conserved motifs and to assess structural variance among serpin proteins. In total, five conserved motifs were identified and designated as motifs 1–5. Among them, motif 1 was the basic hinge motif of the serpin domain, harboring highly conserved sequences (Table 1) which are thought to be involved in the inhibition of proteases [51]. Nearly all serpin proteins contained motifs 1, 2 and 3, with the exception of a few proteins that lacked one or two of the three motifs, whereas high divergence was observed for motif 4 and motif 5. Moreover, serpins within the same clade of the phylogenetic tree contained similar motif arrangements (Figure 2). For instance, the segmentally duplicated genes (BdSRP1-1/BdSRP1-4, HvSRP4-7/HvSRP7-1, HvSRP5-3/HvSRP4-4 and HvSRP5-2/HvSRP4-1) had similar motif compositions and were clustered in their same respective groups within the phylogenetic tree (Figure 2). The same was the case for tandem duplicated genes. This consistency of motif composition of serpin proteins with phylogenetic tree further supported the role of gene duplication events in the diversification and expansion of the serpin gene family in *Brachypodium* and barley.

### 2.3. Subcellular Localization

The subcellular localization analysis indicated that the majority of the BdSRPs (16 out of 27, 59%) are localized in the chloroplast, whereas five proteins were predicted to be localized in the cytoplasm and the remaining six proteins were distributed in the endoplasmic reticulum, mitochondria, nucleus and plasma membrane. The barley serpins showed localization in the cytoplasm, chloroplast, mitochondria and endoplasmic reticulum (Table S1). Subcellular localization studies using GFP- fusion proteins conducted on *Arabidopsis* serpins revealed that GFP-AtSRP2 (At2g14540) was located in the nucleus [26], whereas GFP-AtSRP3 (At1g64030), GFP-ArathZx (At1g47710) and GFP-ArathZ3 (At2g26390) were found in the cytosol [1,26]. Based on immunogold-based localization analysis of *Arabidopsis* serpins, ArathZx was observed in the endoplasmic reticulum, Golgi bodies and cell wall [1]. These data support our findings, in which serpin proteins show diverse subcellular localization. Further work will be required to establish their exact subcellular localization in plants.

The results of SignalP analysis showed that the majority of BdSRPs and HvSRP proteins lack cleavable N-terminal signal sequences and are predicted to be intracellular, suggesting their cytosolic localization, which agrees with previous serpin analysis in plants, where all known serpins exist in intracellular form [27]. However, only two genes from *Brachypodium* (BdSRP4-8 and BdSRP4-10) and one from barley (HvSRP6-1) were predicted to possess a signal peptide at the N-terminal end. In animals, intracellular serpins function mainly in maintaining the uncontrolled proteolytic activity against inflammation and necrosis [2,52]. In *Arabidopsis*, AtSerpin 1 was found to be closely related to intracellular mammalian serpins (Clade B), which lack a recognizable signal peptide at the N-terminal region [2]. However, the function of BdSRP and HvSRP intracellular serpins needs to be verified experimentally.

### 2.4. Duplication and Evolutionary Pattern of Serpin Genes

It is known that tandem duplication and segmental duplication events in plants have been one of the primary driving forces in the evolution and expansion of the gene family and the establishment of new protein functions [53]. Segmental duplication involves duplication between different chromosomes and the same clades, whereas tandem duplication refers to the duplication of two or more genes located on the same chromosome [54]. Gene duplication analysis showed that 19 (19/27; 70%) BdSRP genes were tandemly duplicated, which were recognized on chromosome 4 with five distinct clusters containing 15 genes, whereas chromosome 1 and chromosome 5 contained a single gene pair (Figure 1a). On the other hand, BdSRP1-1 and BdSRP1-4 appeared as segmentally duplicated genes. BdSRP genes without duplicated sequences were thought to have originated from different progenitors. The occurrence of genes in the current study at the same chromosomal location implies a common origin, from which they might have evolved by a series of duplication events. As for *Brachypodium*, 9 out of 25 (36%) HvSRP genes in barley were tandemly duplicated (Figure 1b). Among them, three gene pairs (HvSRP2-2/HvSRP2-3, HvSRP4-2/HvSRP4-3 and HvSRP6-1/HvSRP6-2) were located tandemly at a single locus on chromosome 2H, 4H and 6H respectively, whereas 5H had three tandemly duplicated genes. In addition, the HvSRP4-7/HvSRP7-1, HvSRP5-3/HvSRP4-4 and HvSRP5-2/HvSRP4-1 pairs were segmentally duplicated. In summary, it is likely that tandem and segmental duplications may have played a critical role in the expansion and evolution of the serpin gene family in plants, resulting in their structural and functional diversification.

The molecular evolutionary rate of tandem and segmentally duplicated serpin genes was calculated to explore selective constraints on duplicated serpin genes. The ratio of non-synonymous substitution (K_a_) and synonymous substitution (K_s_) is an effective measure to examine selection pressure among duplicated gene pairs. Therefore, K_a_, K_s_ and K_a_/K_s_ values for each paralogous gene pair was calculated. Generally, K_a_/K_s_ < 1 signifies a strong purifying selection (also called negative selection), whereas K_a_/K_s_ > 1 indicates accelerated evolution with positive selection [55]. Moreover, the value of K_a_/K_s_ = 1 indicates neutral selection. In the current study, 19 genes were found to be tandem duplicates and one segmental duplication gene pair was identified in *Brachypodium*. The K_a_/K_s_ values of BdSRPs ranged from 0.15–0.75, with an average value of 0.48 for tandem duplication genes (Table 2) and 0.42 between a pair of segmentally duplicated genes (Table 3). In barley, the average K_a_/K_s_ values among tandem and segmentally duplicated HvSRP gene pairs were 0.55 (0.29–0.84 range) (Table 4) and 0.28 (0.25–0.30 range), respectively (Table 5). The overall K_a_/K_s_ ratios show that most of the duplicated serpin gene pairs were less than 1, suggesting that these genes may have evolved from intensive purifying selection pressure by natural selection during the evolutionary process.

In addition, divergence periods for segmental and tandem duplicated gene pairs in *Brachypodium* were estimated to have originated approximately 19.23 and 29.36 MYA (million years ago), respectively (Table 2 and Table 3), and the majority of gene pairs were found to have diverged long before the divergence time of grass species (56–73 MYA) [56,57]. In barley, the estimated divergence time was about 25.36 MYA for tandem duplicated genes and 38.56 MYA for segmental duplicated gene pairs (Table 4 and Table 5), in which the divergence of three gene pairs (HvSRP2-2/HvSRP2-3, HvSRP5-3/HvSRP4-4 and HvSRP4-1/HvSRP5-2) appeared to have occurred about 53.74, 55.42 and 56.20 MYA, respectively, which is in close agreement with the divergence time of grasses (56–73 MYA) [57]. Moreover, one gene pair (HvSRP5-2/HvSRP5-3) was estimated to diverge about 2.87 MYA and may represent the newly duplicated gene pair, whereas other gene pairs were estimated to have originated before the divergence of Poaceae. An analysis of the serpin gene family in rice also provides evidence for recent duplication events [9]. Thus, from the above data it can be concluded that the expansion of the serpin gene family in *Brachypodium* and barley can be associated with gene duplication events.

### 2.5. Domain Analysis

**Figure 3:**
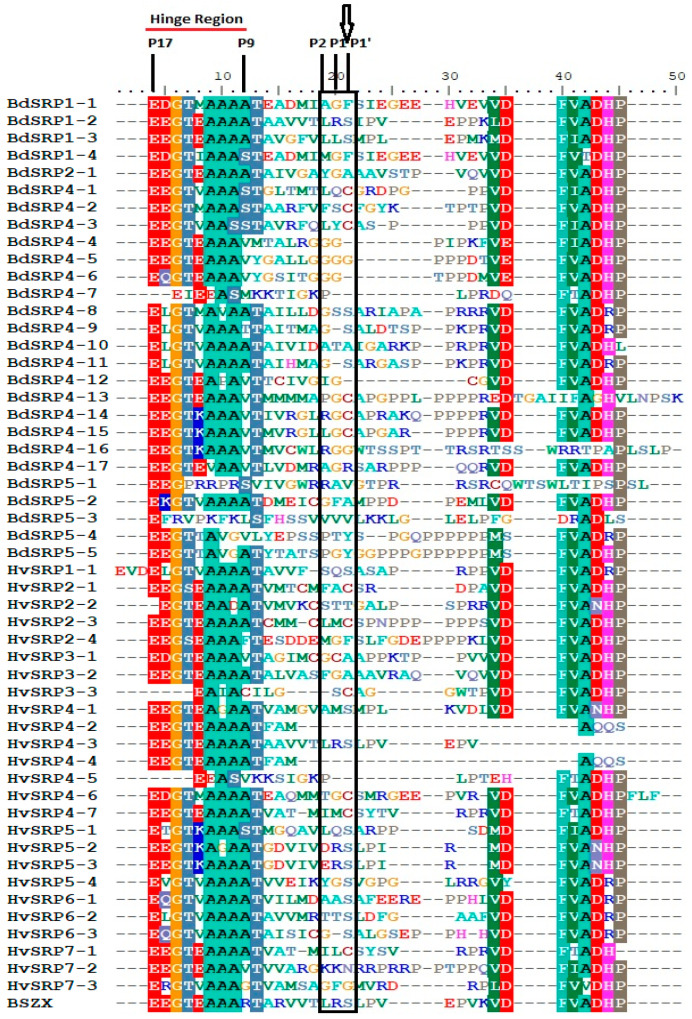
Multiple sequence alignment of the reactive center loop (RCL) of serpin genes in *Brachypodium* (Bd21) and barley. The reactive site residue P1 is marked by an arrow. The conserved hinge region (P9–P17) is underlined.

The multiple sequence alignment of RCL (reactive center loop) regions of BdSRPs and HvSRPs revealed that characteristic residues such as P17 (E, Glu), P15 (G, Gly), P14 (T, Thr) and P8 (T/S, Thr/Ser) in the hinge region are quite conserved in all sequences (Figure 3). However, P2–P1′ sequences were highly variable, with considerable diversity at the critical P1 residue. The residues identified at the P1 position included positively charged residues (Arg and Leu), smaller residues (Gly, Ala, Ser), and hydrophobic (Leu, Met) residues. Compared to barley, the majority of serpins in *Brachypodium* have a small residue (Gly) at the P1 position, whereas the barley P1 residue was highly diversed (Figure 3). Such P1 residue diversity was evident for serpins of rice [9], *Arabidopsis* [1] and oats [58], which may point out a range of inhibitory specificity with disparate functions. Notably, the P17–P9 portion of the RCL (also known as the hinge region) among all serpin genes was found to contain highly conserved consensus EGTEAAAAT sequences (Figure 3), and hence is expected to be indispensable for inhibitory activity [20]. Furthermore, serpins at locus Bradi4g22020 (BdSRP4-7), Bradi5g16744 (BdSRP5-4) and Bradi5g16780 (BdSRP5-5) contain unusual hinge residues instead of the canonical AAAA of inhibitory serpins at P12–P9 positions, and were thus predicted to be non-inhibitory serpins. Among barley serpins, two such non-inhibitory serpins (HvSRP3-3 and HvSRP4-5) were identified that are almost identical to BdSRP4-7, including a deletion of P14 to P17. Based on the presence of Thr at P10 and Val at P11, ArathZ5 was characterized as non-inhibitory in *Arabidopsis* [1]. According to Francis et al. [9] rice has three non-inhibitory serpins (OsSRP-PLP, OsSRP-PTY and OsSRP-PGY) due to unique reactive center residues. Based on the previous example of chaperone-like functions found for barley grain serpins [31], the non-inhibitory capacities of serpins in *Brachypodium* and other plants should be considered for their possible functional roles in plants.

Generally, the most common active site residues of serpins in plants at P2–P1′ are Leu-Arg-Xaa (Xaa = small residue). Plant serpins with such a reactive center are widespread and are known as “LR serpins” [1]. The serpins BSZx [43,59] of barley, ArathZx (AtSerpin1) [22,23] of *Arabidopsis*, OsSRP-LRS (Os03g41419) of rice [9] and Sbser1 (Sb01g014740) [20] of sorghum are well known examples of LR serpins. The results of our study indicate that *Brachypodium* and barley have a single LR serpin gene, namely, BdSRP1-2 (Bradi1g14730) and HvSRP4-3 (HORVU4Hr1G013550) respectively, which complement the LR serpin orthologs of *Arabidopsis* (ArathZx; AtSerpin1), rice (OsSRP-LRS; Os03g41419) and sorghum (Sb01g014740) [9,20,23]. In addition, BdSRP1-2, HvSRP4-2, HvSRP4-3 and HvSRP4-4 were found to share 84%, 90%, 93% and 90% similarity with the previously identified BSZX, respectively (Table S3). These LR serpins were found to be efficient inhibitors of proteinases of different specificity, i.e., proteases with trypsin-like specificity at the canonical P1 Arg and chymotrypsin-like specificity at the canonical P2 Leu [1,9]. The LR serpin genes were used as insect resistance genes and were used to control the programmed cell death in many species [1,19,20,22,24]. For instance, AtSerpin1 inhibits RD21 and thus plays a pro-survival role in relation to excessive cell death due to fungal attack [24]. In addition, AtSerpin1 was found to confer resistance against a wide range of agricultural pests and also inhibited the growth of the cotton leafworm (*Spodoptera littoralis*) when added to the insect diet [19]. Similarly, sorghum LR serpin (Sbser1) was used as an insect resistance against corn earworms [20]. The OsSRP-LRS gene (the closest homolog of AtSerpin1 in rice) codes for an LR serpin in rice that negatively regulates stress-induced cell death [55]. Another LR serpin gene (MtSer6) in *Medicago truncatula* was implicated in the regulation of proteases in order to control proteolysis-dependent cellular damage and nodule senescence under drought-stressed conditions [29]. From these results, it appears that LR serpins share a common function throughout the plant kingdom, due to the highly conserved nature of active site residues [1], which may reflect their role in regulating one or more endogenous proteases in plants. BSZx was shown to be a very potent inhibitor with overlapping reactive centers [58]. Moreover, the most common P1 residue in all plant serpins is Arg (the positively charged residue) and the majority of serpins contain Leu at P2 [1,9,27]. In our analysis, *Brachypodium* has one serpin (BdSRP1-2) with the positively charged residue Arg at P1, whereas barley has three such serpins (HvSRP4-3, HvSRP5-3 and HvSRP7-3). These results suggest that the LR serpins identified in *Brachypodium* and barley in this report can be utilized as a potential candidate for exploitation in disease resistance, pest control and preventing stress-induced cell death.

### 2.6. Synteny and Phylogenetic Analysis

**Figure 4:**
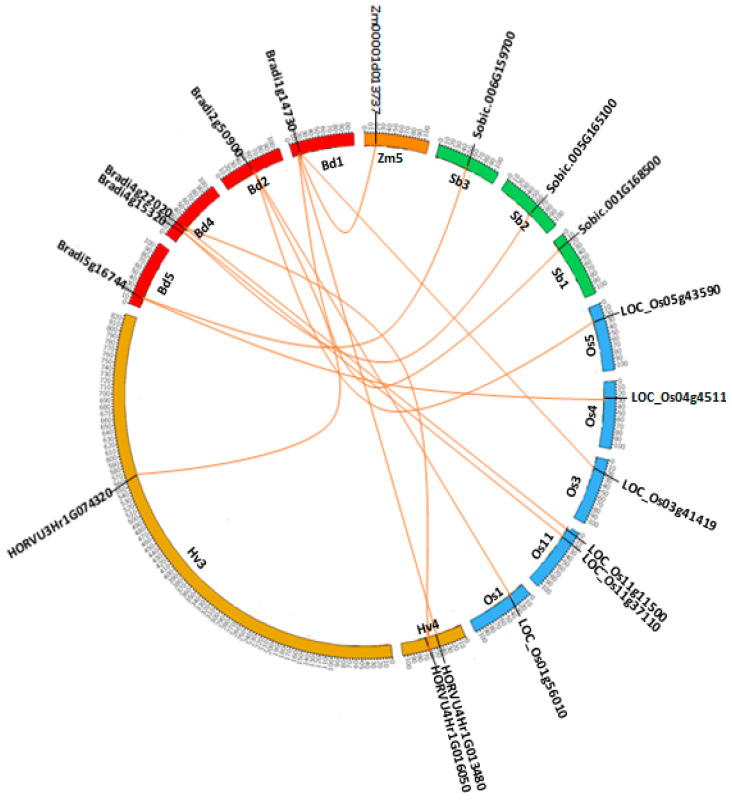
Syntenic block among serpin genes from *Brachypodium*, rice, sorghum, barley and maize. Chromosomes of *Brachypodium* (Bd), rice (Os), sorghum (Sb), barley (Hv) and maize (Zm) are shown in different colors and in circular form. The approximate position of the serpin genes is labeled with a short black line on the circle. Colored curves represented the syntenic relationships between *Brachypodium*, rice, sorghum, barley and maize serpin genes.

Synteny provides a framework in which conservation of homologous genes and gene order is identified between genomes of different species. Therefore, synteny analysis between *Brachypodium* and other grass species was performed to further explore the origin and evolutionary dynamic of the serpin gene family. This analysis revealed that three BdSRPs (Bradi1g14730, Bradi2g50900 and Bradi4g22020) displayed syntenic location to corresponding barley serpin orthologs (HORVU4Hr1G013480, HORVU3Hr1G074320 and HORVU4Hr1G016050) (Figure 4). Although 24 serpin genes in *Brachypodium* could not find their barley orthologs, we could suggest that these orthologous genes evolved after the divergence of *Brachypodium* and barley from their last ancestor. Among synteny events between *Brachypodium* and rice, six gene pairs (Bradi1g14730/LOC_Os03g41419, Bradi2g50900/LOC_Os01g56010, Bradi2g50900/LOC_Os05g43590, Bradi4g15320/LOC_Os11g37110, Bradi4g22020/LOC_Os11g11500, Bradi5g16744/LOC_Os04g45110) showed a syntenic relationship, whereas three syntenic gene pairs were characterized between Bracypodium and sorghum (Bradi1g14730/Sobic.001G168500, Bradi4g15320/Sobic.005G165100, Bradi5g16744/Sobic.006G159700) and only one gene of maize (Zm00001d013737) had a syntenic association with a *Brachypodium* gene (Bradi1g14730) (Figure 4). Intriguingly, one of *Brachypodium* genes (Bradi1g14730, an LR serpin) had orthologs in all analyzed species, like rice (LOC_Os03g41419), barley (HORVU4Hr1G013480), sorghum (Sobic.001G168500) and maize (Zm00001d013737), which may indicate that LR serpins are the ancestral progenitors of all other serpins in plants and thar its function has been retained, as well as its conserved sequence. This synteny analysis among *Brachypodium*, barley, rice, sorghum and maize also revealed that these genes located in syntenic blocks arose before the divergence of these species from a common ancestor. Moreover, it makes a good entry point for clarifying the evolutionary process and retention of the serpin gene family in *Brachypodium* and other grass species. Additionally, the functional characterization of these genes may provide information for serpin homologs in other plant species.

**Figure 5:**
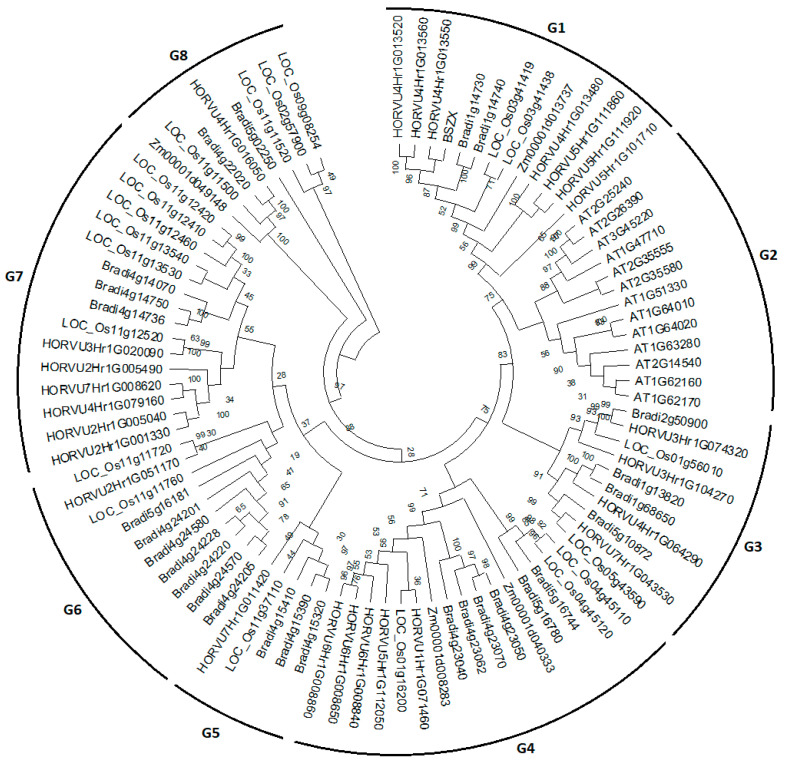
Phylogenetic analysis of serpin genes in *Brachypodium* (Bd21), barley, rice, maize and *Arabidopsis* by MEGA 6.0 software.

To delineate the comparative phylogenetic relationship of BdSRPs and HvSRPs with other plant serpin genes, an unrooted neighbor joining (NJ) phylogenetic tree was constructed by including serpin protein sequences—27 from *Brachypodium* (Bd21), 25 from barley, 20 from rice, 3 from maize and 13 from *Arabidopsis* (Figure 5). BSZX serpin gene (Accession # Q40066) was also included as a reference for comparative phylogenetic analysis. According to the phylogenetic results, a total of eight groups (G1-G8) were recognized. The largest group (G4) included 16 sequences—six from *Brachypodium*, three from rice, five from barley and two from maize. The smallest group was G5, containing only five sequences—three from *Brachypodium* and one each from barley and rice. The other serpin genes from *Brachypodium* and barley, together with rice and maize orthologs, were distributed in G1, G2, G3, G7 and G8. Group 2 was mainly comprised of AtSRPs without any single serpin gene from monocots, suggesting that serpin genes in plants acquired their main diversity after speciation, which closely agrees with the phylogenetic analysis of plant serpin genes by Santamaría et al. [60], in which monocot and eudicot clades were separated, suggesting species-specific (or clade-specific) proliferations. In another phylogenetic analysis of plant serpins, Cohen et al. [61] revealed that the majority of plant serpin are species-specific. Consequently, these serpins might be specifically modified for function according to the need of each particular species. Among all groups in the present report, most of the tandemly duplicated serpin genes from *Brachypodium* and barley were clustered together within a single group, having strong bootstrap values. This is due to the fact that most of the duplicated gene pairs share high amino acid sequence identity and possessed similar exon/intron structures. In addition, *Brachypodium* serpin genes were found to be more closely related to both rice and barley.

### 2.7. Development and Tissue Specific Expression Analysis of Serpin Genes

**Figure 6:**
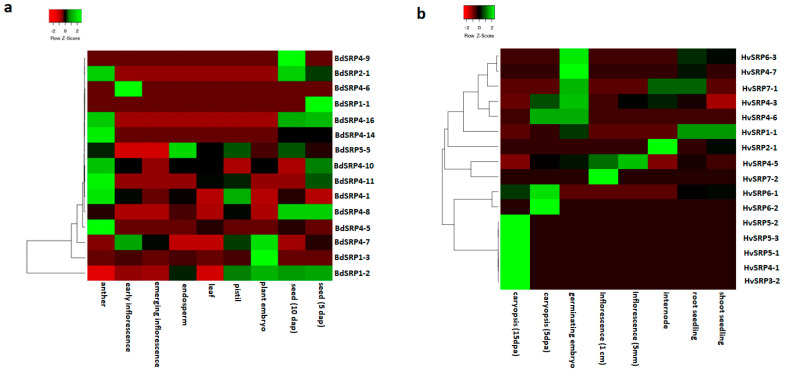
Expression patterns of *Brachypodium* (Bd21) and barley serpin genes in various tissues/organs and developmental stages. Hierarchical clustering analysis of 15 BdSRP genes (**a**) and 16 HvSRP genes (**b**) were based on Pearson’s correlation.

Expression data for *Brachypodium* and barley in various tissues and developmental stages was obtained by using publically available gene expression databases. Among all BdSRPs, the expression data of only 15 genes was found, and many of these genes showed a distinct tissue-specific expression, indicating a specific role in particular stages of development. For instance, the expression of BdSRP4-10, BdSRP4-11, BdSRP4-16 and BdSRP2-1 were preferentially high both in the seed and anther (Figure 6a). Similarly, the expression of two genes (BdSRP4-1, BdSRP4-5) was restricted to the anther only, whereas BdSRP1-3 and BdSRP4-7 were preferentially expressed in the embryo. BdSRP5-5 showed significant transcript accumulation in the endosperm, pistil and seed at 5 days post-anthesis (dpa). Notably, BdSRP1-2 was a comparatively highly expressed gene, showing expression during seed development (5 and 10 dpa), pistil and embryo, which is suggestive of its broad role in plant development. It should be noted that the anatomy of *Brachypodium* and cereal seeds are different. *Brachypodium* has much less starch (10%) compared to cereal grains (50–70%), which further adds to a species-specific adaptation of serpins, keeping the relation between protein z and β-amylase in barley in mind. In wheat seeds, an association between amyloplast and serpins has recently been found [62]. A similar analysis was performed for rice serpin genes using microarray data [9], in which a high level of expression was observed for OsSRP-LRS (Os03g41419), OsSRP-PLP (Os11g11500), OsSRP-FRS (Os03g41438) and OsSRP-LGC (Os01g56010) at different developmental stages. In *Arabidopsis*, a substantial level of basal expression was detected for six serpin coding genes in developing seedlings and mature tissues [26]. In wheat, a majority of serpin genes (55 genes) were expressed in the grain or spike of wheat during grain filling [36], which also reflects the polyploidy nature of wheat.

The expression analysis performed for barley serpin genes (Figure 6b) indicated that seven genes (HvSRP3-2, HvSRP4-1, HvSRP5-1, HvSRP5-2, HvSRP5-3, HvSRP6-1, HvSRP6-2) out of 25 were highly expressed in caryopsis (15 dpa). A diverse expression pattern for HvSRP1-1 was observed in the root seedling, shoot seedling and internodal area. Likewise, HvSRP7-1 exhibited a broad expression in the root seedling and internodes and embryo, whereas the expression of HvSRP4-3, HvSRP4-7 and HvSRP6-3 was confined to the germinating embryo only. HvSRP4-6 was expressed at a high rate in the caryopsis and embryo. These data support an earlier study on barley serpin genes, wherein a high expression was reported for HorvuZ4 (BSZ4) in both caryopsis (16 dap sample) and endosperm (26 dap sample). In contrast, the expression of HorvuZx was observed in almost all tissues [27]. Proteomic experiments in rice confirmed the expression of OsSRP-LRS in the seed and OsSRP-LGC in the root [63]. From the above data, it can be proposed that the seeds abundant in serpins may have a protective role against insects and pathogens, supplementing their utility in grain development, whereas other serpins may be involved in regulating programmed cell death. Thus, serpin genes are interesting targets for characterization and breeding in plants for defense against plant pathogens and grain development.

## 3. Materials and Methods

### 3.1. Sequence Analysis

Serpin protein and gene sequences for *Brachypodium* (*Brachypodium distachyon*) ecotype Bd21 (https://plants.ensembl.org/Brachypodium_distachyon/Info/Annotation/) and barley (*Hordeum vulgare*, cv. Morex) (http://plants.ensembl.org/Hordeum_vulgare/Info/Index) were extracted from the Plaza 4.5 database (http://bioinformatics.psb.ugent.be/plaza/) and Ensembl Plants (https://plants.ensembl.org/index.html). For this, a BLASTP search was performed against barley and *Brachypodium* genomes using the barley serpin gene sequence (Accession # Q40066) as a query. The ecotype Bd21-3 genome [43] was also used for the comparison of gene-copy numbers, using the BrachyPan database (https://brachypan.jgi.doe.gov/). Subcellular localization of serpin genes was predicted using WoLF PSORT [64] and signal peptides were predicted using SignalP 5.0 (http://www.cbs.dtu.dk/services/SignalP/). The theoretical molecular weight (Mol. wt) and isoelectric point (pI) values were predicted using the ProtParam tool (http://web.expasy. org/protparam).

### 3.2. Determination of Chromosomal Location and Synteny Analysis

Chromosomal locations, and sizes (bp) of serpin genes were obtained from the Plaza 4.5 database (https://bioinformatics.psb.ugent.be/plaza/) and were used to map on respective chromosomes using the MapChart software (https://www.wur.nl/en/show/Mapchart.htm). Tandemly duplicated serpin genes in the *Brachypodium* and barley genomes were defined as adjacent to homologous serpin genes on chromosomes or within a sequence distance of 50 kb [65]. For synteny analysis, syntenic blocks between *Brachypodium* and barley, maize, rice and sorghum genomes containing serpin genes were downloaded from the Plant Genome Duplication database (http://chibba.pgml.uga.edu/duplication/) and visualized (including gene locations) using Circos software (http://circos.ca/) [66].

### 3.3. Gene Structure and Conserved Motif Identification

Intron/exon sites of serpin genes were investigated using Gene Structure Display Server v2.0 (GSDS, http://gsds.cbi.pku.edu.cn/). For this, coding sequences were compared to their corresponding genomic sequences in GSDS. Thereafter, these sequences were submitted to the Multiple EM for Motif Elicitation tool (MEME v4.11.1; http://meme-suite.org/) [67] to identify conserved motifs among serpin proteins.

### 3.4. Alignment of Sequences and Phylogenetic Analysis

The amino sequences of serpin genes were aligned using the Clustal W program. The phylogenetic tree was constructed based on this alignment using an NJ (neighbor-joining) phylogram in MEGA 6 software [68] with 1000 replicates.

### 3.5. Evolutionary Rate Calculations

To estimate the molecular evolutionary rates of duplicated gene pairs, the non-synonymous substitution (K_a_) and synonymous substitution (K_s_) rate ratios of ortholog gene pairs of serpins were calculated using the Codeml program in the PAML v4.3 [69] interface tool of PAL2NAL [70] after aligning amino acid sequences and the corresponding nucleotide sequences. Based on a rate of 6.1 × 10^−9^ substitutions per site per year, we calculated the divergence time (T) as T = K_s_/(2 × 6.1 × 10^−9^) × 10^−6^ Mya for *Brachypodium* and barley [55].

### 3.6. Database Search for Expression Data of Serpin Genes

The expression patterns of serpin genes during various developmental stages were also analyzed using Gene Expression Atlas of EMBL-EBI (http://www. ebi.ac.uk/gxa/) for *Brachypodium* and barley. Heat maps were generated using the heatmapper online tool (http://www.heatmapper.ca/expression/).

## 4. Conclusions

In our study, the identification, phylogeny, domain structure and comparative analysis of the serpin gene family were carried out for *Brachypodium* and barley. *Brachypodium* and barley are unusual in that they have twice the number of serpin genes compared to other species; i.e., 27 and 25 serpin encoding genes were identified in *Brachypodium* and barley, respectively. Based on sequence analysis, three genes (BdSRP4-7, BdSRP5-4 and BdSRP5-5) in *Brachypodium* and two genes (HvSRP3-3 and HvSRP4-5) in barley were predicted to be non-inhibitory serpins due to unique reactive center residues, whereas all remaining residues were inhibitory. The existence of non-inhibitory serpins in *Brachypodium* and barley may reflect their possible role in protein storage and chaperone-like functions. The diversity of reactive center sequences in inhibitory serpins indicated a range of inhibitory specificity with disparate functions. Additionally, *Brachypodium* and barley have a single LR serpin gene, namely, BdSRP1-2 and HvSRP4-3 respectively, which is complementary with orthologous genes of *Arabidopsis* (AtSerpin1) and rice (OsSRP-LRS), and are expected to be active in regulating one or more endogenous proteases. The conserved domain and common motifs were predicted and analyzed. Phylogenetic comparisons of serpin genes strongly suggested that the high rate of retention of gene duplication may have resulted in the expansion and functional diversification of these proteins. Expression patterns among different tissues signify their specific roles at different developmental stages. Overall, this research has contributed to the understanding of the serpin gene family in *Brachypodium* and barley, which could be useful for the discovery of new serpin genes from other plants. However, there is enormous scope for further studies on functional information of these serpins in *Brachypodium* and barley.

## Figures and Tables

**Table 1:** Motifs found in the serpin proteins.

Motifs	Sequences
1	RPLYVSSVFHKAVVEVBEEGTEAAAATAA
2	FVADHPFLFLIVEEVSGAVLF
3	DTRLVLGNALYFKGKWTEPFD
4	FSMYIFLPDARDGLWGLADKJ
5	IACHDGFKVLKLPYKQ

**Table 2:** The non-synonymous substitution rate (K_a_) and synonymous substitution rate (K_s_) values and estimated divergence time for tandemly duplicated BdSRP genes. MYA, million years ago.

Segment Pair	K_a_	K_s_	K_a_/K_s_	Estimated Time (MYA)	Mode of Duplication
BdSRP1-2/BdSRP1-3	0.0842	0.3685	0.22846	30.19	Tandem
BdSRP4-5/BdSRP4-6	0.2022	0.3732	0.54167	30.59	Tandem
BdSRP4-5/BdSRP4-4	0.175	0.7633	0.2292	62.56	Tandem
BdSRP4-8/BdSRP4-10	0.2806	0.6012	0.46676	49.27	Tandem
BdSRP4-9/BdSRP4-11	0.0883	0.223	0.39612	18.27	Tandem
BdSRP4-12/BdSRP4-13	0.2209	0.2929	0.75396	24	Tandem
BdSRP4-15/BdSRP4-16	0.0755	0.1509	0.50059	12.36	Tandem
BdSRP4-14/BdSRP4-17	1.0413	0.1591	0.1528	13.04	Tandem
BdSRP5-4/BdSRP5-5	0.3304	0.2925	1.12963	23.97	Tandem
Mean	0.3659	0.3582	0.4887	29.3611	

**Table 3:** The K_a_ and K_s_ values and estimated divergence time for segmentally duplicated BdSRP genes.

Segment Pair	K_a_	K_s_	K_a_/K_s_	Estimated Time (MYA)	Mode of Duplication
BdSRP1-1/BdSRP1-4	0.0986	0.2347	0.42019	19.23	Segmental

**Table 4:** The K_a_ and K_s_ values and estimated divergence time for tandemly duplicated HvSRP genes.

Segment Pair	K_a_	K_s_	K_a_/K_s_	Estimated Time (MYA)	Mode of Duplication
HvSRP2-2/HvSRP2-3	0.1906	0.6556	0.2906	53.7454	Tandem
HvSRP4-2/HvSRP4-3	0.0536	0.0633	0.8479	5.1888	Tandem
HvSRP5-2/HvSRP5-3	0.0193	0.035	0.5513	2.8756	Tandem
HvSRP6-1/HvSRP6-2	0.2058	0.4277	0.4812	35.0602	Tandem
HvSRP6-3/HvSRP6-2	0.2133	0.3655	0.5836	29.9666	Tandem
Mean	0.1365	0.3094	0.5509	25.3673	

**Table 5:** The K_a_ and K_s_ values and estimated divergence time for segmentally duplicated HvSRP genes.

Segment pair	K_a_	K_s_	K_a_/K_s_	Estimated time (MYA)	Mode of duplication
HvSRP4-1/HvSRP5-2	0.2072	0.6857	0.3022	56.2082	Segmental
HvSRP4-4/HvSRP5-3	0.1703	0.6761	0.2518	55.4252	Segmental
HvSRP4-7/HvSRP7-1	0.0146	0.0495	0.2948	4.0591	Segmental
Mean	0.1307	0.469	0.2829	38.5641	

## Supplementary Materials

The following are available online at https://www.mdpi.com/2223-7747/9/11/1439/s1, Table S1: The gene identification numbers of serpin genes in *Brachypodium* and barley, along with length of amino acid sequence (aa), Mol.wt (Dalton), isoelectric point (pI), subcellular localization, putative function and signal peptide (+/-); Table S2: Serpin names for *Arabidopsis*, rice and barley proposed by Robert and Hejgaard (2008); Table S3: The percentage identity observed between serpin genes in *Brachypodium* (Bd21) and barley. BSZX serpin gene was included as a reference for comparative analysis.
