# Supplementary material for: Genome Wide Identification and Comparative Analysis of the Serpin Gene Family in Brachypodium and Barley
Source: Plants (Basel). 2020 Oct 26;9(11):1439. doi: 10.3390/plants9111439 (PMC7692276; doi:10.3390/plants9111439)
Supplement: Supplementary file 1 [file plants-09-01439-s001.zip › plants-971398-supplementart-xml/plants-971398-table S1 S2-xml.docx]

**Table S1.** Serpins names for Arabidopsis, rice and barley proposed by Robert and Hejgaard (2008).

| **Locus name/Accession** | **Serpin names based on Nomenclature (Five letter code for species) proposed by Robert and Hejgaard (2008)** | **RCL Sequence**  **(P2-P1′ in bold)** |
| --- | --- | --- |
| At1g47710 | ArathZx | P14 P8 P1 P2’  \| \| \| \|  EEGTEAAAASAGVIK**LRG**LLM—EEDEIDF |
| At1g62170 | ArathZ5 | \| \| \| \|  EKGTEAVTFTAFRSA**YLG**CAL---VKPIDF |
| At1g64030 | ArathZ1 | \| \| \| \|  EEGAEAAAATADGDC**GCS**LDFVEPPKKIDF |
| At2g14540 | ArathZ2 | \| \| \| \|  EEGTEAAAATTVVVV**TGS**CLW-EPKKKIDF |
| At2g25240 | ArathZ10 | \| \| \| \|  EEGTEAAAVSVGVVS**CTS**FRR-----NPDF |
| At2g26390 | ArathZ3 | \| \| \| \|  EEGTEAAAVSVAIMM**PQC**LMR-----NPDF |
| At3g45220 | ArathZ4 | \| \| \| \|  EEGTEAAAVSVASMT**KDM**LLM------GDF |
| Os01g16200 | OrysaZ12 | \| \| \| \|  ELGTVAAASTAVVM**MQK**GSSL------PPVDF |
| Os01g56010 | OrysaZ1 | \| \| \| \|  EEGTEAAAATAVVMT**LGC**AAP—SAPVHVVDF |
| Os11g12520 | OrysaZ5 | \| \| \| \|  EEGTEAAASAINMVC**GMS**MTP--EPRPVPVDF |
| Os11g12410 | OrysaZ6a | \| \| \| \|  EEGTEAAAATAVLME**GAA**RYA--PPPPPREDF |
| Os04g45110 | OrysaZ10 | \| \| \| \|  EEGTTAVEATYSCCS**PTY**SGP-ESPKPRPMSF |
| Os04g45120 | OrysaZ11 | \| \| \| \|  EEGTTAVEAMYSPSS**PGY**SPGYQPPRPPPMSF |
| Os11g11500 | OrysaZ9 | \| \| \| \|  QKGIEETSVSMGLGK**PLP**AQH----------F |
| Os11g11760 | OrysaZ8 | \| \| \| \|  EEGTVAAAATMTRML**PSG**VPPPPVDFVAEHPF |
|  |  |  |
| Os11g12420 | OrysaZ6b | \| \| \| \|  EEGTEVAAATVVIMK**GRA**RRP--SPAPAPVDF |
| Os11g12460 | OrysaZ6c | \| \| \| \|  EEGTEAAAATAVCLT**FAS**AAP-SSRRPARVDF |
| Os11g13530 | OrysaZ2a | \| \| \| \|  EEGTEAAASTACTIR**LLS**MSY-------PEDF |
| Os11g13540 | OrysaZ2b | \| \| \| \|  EEGTEAAAATACTMK**FLC**LTL-----TSPVDF |
| Os03g41419b | OrysaZxa | \| \| \| \|  EEGTEAAAATAAVIT**LRS**API-------AEDF |
| Os03g41438 | OrysaZxb | \| \| \| \|  EEGTEAAAASAAVVS**FRS**APV-------TVDF |
| BSZ7_HORVU/ Q43492 | HorvuZ7 | \| \| \| \|  EEGTKAGAATGDVIV**DRS**LPIRMDFVA |
| SPZX_HORVU/ Q40066 | HorvuZx | \| \| \| \|  EEGTEAAARTARVVT**LRS**LPVEPVKVD |
| SPZ4_HORVU / P06293.2 | HorvuZ4 | \| \| \| \|  EEGTEAGAATVAMGVA**MSM**PLKVDLVD |

**Table S2.** The gene identification numbers of serpin genes in Brachypodium and barley along with length of amino acid sequence (aa), Mol.wt (Dalton), isoelectric point (pI), subcellular localization, putative function and signal peptide (+/-).

| **Gene Name** | **Gene ID** | **Genome** | **Location** | **Protein** | | | **No. of Introns** | **Sub Cellular Localization** | **Putative function** | **Signal peptide** |
| --- | --- | --- | --- | --- | --- | --- | --- | --- | --- | --- |
|  |  |  |  | **Length (aa)** | **Mol.wt (kDa)** | **pI** |  |  |  |  |
| **BdSRP1-1** | **Bradi1g13820** | ***Brachypodium distachyon Bd21*** | **Bd1 : 10663802-10665142** | **391** | **42.71** | **5.09** | 1 | Endoplasmic reticulum/Nucleus | Inhibitory | - |
| BdSRP1-2 | Bradi1g14730 | *Brachypodium distachyon Bd21* | Bd1 : 11672959-11675271 | 401 | 42.66 | 6.38 | 1 | Chloroplast | Inhibitory | - |
| BdSRP1-3 | Bradi1g14740 | *Brachypodium distachyon Bd21* | Bd1 : 11678289-11680572 | 403 | 42.69 | 5.17 | 1 | Chloroplast | Inhibitory | - |
| BdSRP1-4 | Bradi1g68650 | *Brachypodium distachyon Bd21* | Bd1 : 67400987-67402283 | 393 | 43.05 | 4.89 | 1 | Chloroplast | Inhibitory | - |
| BdSRP2-1 | Bradi2g50900 | *Brachypodium distachyon Bd21* | Bd2 : 50390802-50392082 | 399 | 42.65 | 6.53 | 1 | Chloroplast | Inhibitory | - |
| BdSRP4-1 | Bradi4g14070 | *Brachypodium distachyon Bd21* | Bd4 : 14583397-14585085 | 460 | 49.86 | 7.12 | 2 | Chloroplast | Inhibitory | - |
| BdSRP4-2 | Bradi4g14736 | *Brachypodium distachyon Bd21* | Bd4 : 15357919-15365758 | 439 | 48.07 | 7.19 | 5 | Chloroplast | Inhibitory | - |
| BdSRP4-3 | Bradi4g14750 | *Brachypodium distachyon Bd21* | Bd4 : 15376209-15377202 | 313 | 34.99 | 9.01 | 1 | Endoplasmic reticulum | Inhibitory | - |
| BdSRP4-4 | Bradi4g15320 | *Brachypodium distachyon Bd21* | Bd4: 16020814-16021056 | 80 | 8.66 | 5.19 | 0 | Cytoplasm | Inhibitory | - |
| BdSRP4-5 | Bradi4g15390 | *Brachypodium distachyon Bd21* | Bd4 : 16087714-16089082 | 336 | 36.83 | 6.64 | 1 | Cytoplasm | Inhibitory | - |
| BdSRP4-6 | Bradi4g15410 | *Brachypodium distachyon Bd21* | Bd4 : 16110335-16112008 | 317 | 33.63 | 8.52 | 3 | Chloroplast | Inhibitory | - |
| BdSRP4-7 | Bradi4g22020 | *Brachypodium distachyon Bd21* | Bd4 : 25874867-25876705 | 394 | 42.82 | 5.83 | 2 | Mitochondria | Non-Inhibitory | - |
| BdSRP4-8 | Bradi4g23040 | *Brachypodium distachyon Bd21* | Bd4 : 27487566-27488903 | 445 | 48.82 | 10.04 | 0 | Chloroplast | Inhibitory | + |
| BdSRP4-9 | Bradi4g23050 | *Brachypodium distachyon Bd21* | Bd4 : 27491658-27492806 | 382 | 41.28 | 6.20 | 0 | Chloroplast | Inhibitory | - |
| BdSRP4-10 | Bradi4g23062 | *Brachypodium distachyon Bd21* | Bd4 : 27541712-27543002 | 402 | 43.44 | 8.77 | 2 | Chloroplast | Inhibitory | + |
| BdSRP4-11 | Bradi4g23070 | *Brachypodium distachyon Bd21* | Bd4 : 27544036-27545559 | 507 | 54.88 | 9.69 | 1 | Chloroplast | Inhibitory | - |
| BdSRP4-12 | Bradi4g24201 | *Brachypodium distachyon Bd21* | Bd4 : 29220825-29222233 | 444 | 48.04 | 6.28 | 3 | Plasma membrane | Inhibitory | - |
| BdSRP4-13 | Bradi4g24205 | *Brachypodium distachyon Bd21* | Bd4 : 29228278-29229963 | 540 | 57.76 | 8.33 | 2 | Chloroplast | Inhibitory | - |
| BdSRP4-14 | Bradi4g24220 | *Brachypodium distachyon Bd21* | Bd4 : 29279220-29280339 | 325 | 35.94 | 9.71 | 1 | Cytoplasm | Inhibitory | - |
| BdSRP4-15 | Bradi4g24228 | *Brachypodium distachyon Bd21* | Bd4 : 29282544-29283789 | 384 | 41.20 | 9.32 | 1 | Chloroplast | Inhibitory | - |
| BdSRP4-16 | Bradi4g24570 | *Brachypodium distachyon Bd21* | Bd4 : 29678395-29679577 | 355 | 38.49 | 8.49 | 2 | Chloroplast | Inhibitory | - |
| BdSRP4-17 | Bradi4g24580 | *Brachypodium distachyon Bd21* | Bd4 : 29685641-29687167 | 508 | 55.58 | 4.95 | 0 | Nuclear | Inhibitory | - |
| BdSRP5-1 | Bradi5g02250 | *Brachypodium distachyon Bd21* | Bd5 : 2395906-2396743 | 272 | 29.33 | 5.91 | 2 | Endoplasmic reticulum | Inhibitory | - |
| BdSRP5-2 | Bradi5g10872 | *Brachypodium distachyon Bd21* | Bd5 : 14378527-14379806 | 412 | 44.13 | 4.85 | 1 | Cytoplasm | Inhibitory | - |
| BdSRP5-3 | Bradi5g16181 | *Brachypodium distachyon Bd21* | Bd5 : 19730271-19733240 | 358 | 38.36 | 9.51 | 1 | Chloroplast | Inhibitory | - |
| BdSRP5-4 | Bradi5g16744/KQJ83765 | *Brachypodium distachyon Bd21* | Bd5 : 20179381-20179875 | 164 | 17.50 | 4.32 | 0 | Cytoplasmic | Non-Inhibitory | - |
| BdSRP5-5 | Bradi5g16780 | *Brachypodium distachyon Bd21* | Bd5 : 20219909-20221254 | 406 | 44.13 | 6.66 | 1 | Chloroplast | Non-Inhibitory | - |
| BdSRP1-1 | BdiBd21-3.1G0184000/1G13820 | *Brachypodium distachyon Bd21-3* | Bd1:10813998-10815325 | 352 | 38.60 | 5.42 | 3 | Cytoplasm | Inhibitory | - |
| BdSRP1-2 | BdiBd21-3.1G0197200.1/14730 | *Brachypodium distachyon Bd21-3* | Bd1:11827013-11829942 | 401 | 42.66 | 6.38 | 1 | Mitochondria | Inhibitory | - |
| BdSRP1-3 | BdiBd21-3.1G0197300/14740 | *Brachypodium distachyon Bd21-3* | Bd1:11832656-11835296 | 403 | 42.69 | 5.17 | 1 | Chloroplast | Inhibitory | - |
| BdSRP1-4 | BdiBd21-3.1G0925700/1G68650. | *Brachypodium distachyon Bd21-3* | Bd1:67866200-67867496 | 393 | 43.05 | 4.89 | 1 | Chloroplast | Inhibitory | - |
| BsSRP2-1 | BdiBd21-3.2G0650900/2G50900 | *Brachypodium distachyon Bd21-3* | Bd2:50101565-50103224 | 399 | 42.41 | 6.46 | 1 | Chloroplast | Inhibitory | - |
| BdSRP4-1 | BdiBd21-3.4G0208100/14070 | *Brachypodium distachyon Bd21-3* | Bd4:14658601-14660706 | 460 | 49.77 | 8.55 | 2 | Chloroplast | Inhibitory | - |
| BdSRP4-2 | BdiBd21-3.4G0224500/15320 | *Brachypodium distachyon Bd21-3* | Bd4:15973524-15973766 | 80 | 8.66 | 5.19 | 0 | Cytoplasm | Inhibitory | - |
| BdSRP4-3 | BdiBd21-3.4G0225300/4G15390 | *Brachypodium distachyon Bd21-3* | Bd4:16040205-16041989 | 336 | 36.70 | 6.46 | 1 | Cytoplasm | Inhibitory | - |
| BdSRP4-4 | BdiBd21-3.4G022550/4G15410 | *Brachypodium distachyon Bd21-3* | Bd4:16063047-16064720 | 391 | 41.70 | 7.14 | 2 | Nucleolus | Inhibitory | - |
| BdSRP4-5 | BdiBd21-3.4G0307000/22020 | *Brachypodium distachyon Bd21-3* | Bd4:25910594-25914314 | 463 |  |  | 2 | Mitochondria | Non-Inhibitory | - |
| BdSRP4-6 | BdiBd21-3.4G0320800/4G23040 | *Brachypodium distachyon Bd21-3* | Bd4:27526091-27527428 | 445 | 48.82 | 10.04 | 0 | Chloroplast | Inhibitory | + |
| BdSRP4-7 | BdiBd21-3.4G0320900/4G23050 | *Brachypodium distachyon Bd21-3* | Bd4:27530183-27531331 | 382 | 41.28 | 6.20 | 0 | Chloroplast | Inhibitory | - |
| BdSRP4-8 | BdiBd21-3.4G0321200/4G23062 | *Brachypodium distachyon Bd21-3* | Bd4:27580443-27581733 | 402 | 43.44 | 8.77 | 2 | Chloroplast | Inhibitory | + |
| BdSRP4-9 | BdiBd21-3.4G0321300/ | *Brachypodium distachyon Bd21-3* | Bd4:27582327-27584306 | 285 | 31.14 | 7.07 | 0 | Cytoplasm | Inhibitory | - |
| BdSRP4-10 | BdiBd21-3.4G0340200/4G24201 | *Brachypodium distachyon Bd21-3* | Bd4:29367697-29369105 | 444 | 48.04 | 6.28 | 3 | Plasma membrane | Inhibitory | - |
| BdSRP4-11 | BdiBd21-3.4G0340400/4G24205 | *Brachypodium distachyon Bd21-3* | Bd4:29375150-29376835 | 534 | 56.95 | 8.63 | 2 | Chloroplast | Inhibitory | - |
| BdSRP4-12 | BdiBd21-3.4G0341000/24220 | *Brachypodium distachyon Bd21-3* | Bd4:29426090-29429078 | 531 | 58.05 | 10.76 | 4 | Chloroplast | Inhibitory | - |
| BdSRP4-13 | BdiBd21-3.4G0341100/4G24228 | *Brachypodium distachyon Bd21-3* | Bd4:29429414-29430659 | 384 | 41.20 | 9.32 | 1 | Chloroplast | Inhibitory | - |
| BdSRP4-14 | BdiBd21-3.4G0346400/24570 | *Brachypodium distachyon Bd21-3* | Bd4:29857889-29859071 | 355 | 38.49 | 8.49 | 2 | Chloroplast | Inhibitory | - |
| BdSRP4-15 | BdiBd21-3.4G0346700/4G24580 | *Brachypodium distachyon Bd21-3* | Bd4:29865129-29866650 | 491 | 53.63 | 4.89 | 1 | Nucleolus | Inhibitory | - |
| BdSRP5-1 | BdiBd21-3.5G0028300/5G02250 | *Brachypodium distachyon Bd21-3* | Bd5:2400066-2400903 | 272 | 29.24 | 6.10 | 2 | Endoplasmic reticulum | Inhibitory | - |
| BdSRP5-2 | BdiBd21-3.5G0136100/5G10872 | *Brachypodium distachyon Bd21-3* | Bd5:14462623-14463902 | 412 | 44.13 | 4.85 | 1 | Cytoplasm | Inhibitory | - |
| BdSRP5-3 | BdiBd21-3.5G0210100/5G16181 | *Brachypodium distachyon Bd21-3* | Bd5:19843101-19846070 | 358 | 38.36 | 9.51 | 1 | Chloroplast | Inhibitory | - |
| BdSRP5-4 | BdiBd21-3.5G0218600/5G16744 | *Brachypodium distachyon Bd21-3* | Bd5:20361846-20363616 | 164 | 17.50 | 4.32 | 0 | Chloroplast | Non-Inhibitory | - |
| BdSRP5-5 | BdiBd21-3.5G0219500/16780 | *Brachypodium distachyon Bd21-3* | Bd5:20404124-20405824 | 395 | 42.91 | 6.71 | 1 | Chloroplast | Non-Inhibitory | - |
| HvSRP1-1 | HORVU1Hr1G071460 | *Hordeum vulgare* | Chr1H: 493269717-493271575 | 520 | 55.29 | 6.15 | 0 | Vacuole | Inhibitory | - |
| HvSRP2-1 | HORVU2Hr1G001330 | *Hordeum vulgare* | chr2H:2828084-2829672 | 421 | 45.80 | 6.54 | 1 | Cytoplasm | Inhibitory | - |
| HvSRP2-2 | HORVU2Hr1G005040 | *Hordeum vulgare* | chr2H:11174012-11175402 | 242 | 27.14 | 8.61 | 0 | Cytoplasm | Inhibitory | - |
| HvSRP2-3 | HORVU2Hr1G005490 | *Hordeum vulgare* | chr2H:11949639-11950638 | 305 | 34.07 | 6.06 | 0 | Cytoplasm | Inhibitory | - |
| HvSRP2-4 | HORVU2Hr1G051170 | *Hordeum vulgare* | chr2H:299270641-299271562 | 283 | 32.11 | 5.15 | 0 | Chloroplast | Inhibitory | - |
| HvSRP3-1 | HORVU3Hr1G020090 | *Hordeum vulgare* | chr3H:60856203-60857988 | 492 | 53.51 | 8.08 | 1 | Chloroplast | Inhibitory | - |
| HvSRP3-2 | HORVU3Hr1G074320 | *Hordeum vulgare* | chr3H:558020676-558022073 | 415 | 44.46 | 7.94 | 1 | Mitochondria | Inhibitory | - |
| HvSRP3-3 | HORVU3Hr1G104270 | *Hordeum vulgare* | chr3H:666288585-666289084 | 150 | 16.41 | 5.08 | 0 | Cytoplasm | Non-inhibitory | - |
| HvSRP4-1/Z4 | HORVU4Hr1G013480 | *Hordeum vulgare* | chr4H:47133356-4713508 | 447 | 48.45 | 6.61 | 1 | Cytoplasm | Inhibitory | - |
| HvSRP4-2/ZX | HORVU4Hr1G013520 | *Hordeum vulgare* | chr4H:47765330-47766124 | 223 | 24.86 | 8.62 | 0 | Cytoplasm | Inhibitory | - |
| HvSRP4-3/ZX | HORVU4Hr1G013550 | *Hordeum vulgare* | chr4H:47808720-47811193 | 360 | 30.98 | 9.24 | 3 | Mitochondria | Inhibitory | - |
| HvSRP4-4/ZX | HORVU4Hr1G013560 | *Hordeum vulgare* | chr4H:47897059-47897853 | 223 | 24.86 | 8.62 | 0 | Cytoplasm | Inhibitory | - |
| HvSRP4-5 | HORVU4Hr1G016050 | *Hordeum vulgare* | chr4H:64666800-64678780 | 440 | 47.73 | 8.20 | 2 | Mitochondria | Non-inhibitory | - |
| HvSRP4-6 | HORVU4Hr1G064290 | *Hordeum vulgare* | chr4H:539305293-539306584 | 390 | 42.54 | 5.30 | 1 | Endoplasmic reticulum | Inhibitory | - |
| HvSRP4-7 | HORVU4Hr1G079160 | *Hordeum vulgare* | chr4H:611364147-611365731 | 438 | 47.10 | 5.71 | 2 | Chloroplast | Inhibitory | - |
| HvSRP5-1 | HORVU5Hr1G101710 | *Hordeum vulgare* | chr5H:613035051-613037353 | 277 | 30.98 | 6.12 | 1 | Cytoplasm | Inhibitory | - |
| HvSRP5-2/Z7 | HORVU5Hr1G111860 | *Hordeum vulgare* | chr5H:637402375-637403157 | 260 | 28.81 | 5.06 | 0 | Cytoplasm | Inhibitory | - |
| HvSRP5-3/Z7 | HORVU5Hr1G111920 | *Hordeum vulgare* | chr5H:637647948-637652536 | 430 | 46.64 | 6.47 | 1 | Cytoplasm | Inhibitory | - |
| HvSRP5-4 | HORVU5Hr1G112050 | *Hordeum vulgare* | chr5H:638078313-638079620 | 191 | 21.05 | 5.83 | 0 | Chloroplast | Inhibitory | - |
| HvSRP6-1 | HORVU6Hr1G008650 | *Hordeum vulgare* | chr6H:15815806-15817433 | 438 | 47.17 | 6.33 | 0 | Cytoplasm | Inhibitory | + |
| HvSRP6-2 | HORVU6Hr1G008840 | *Hordeum vulgare* | chr6H:16081808-16083443 | 409 | 44.54 | 9.60 | 0 | Chloroplast | Inhibitory | - |
| HvSRP6-3 | HORVU6Hr1G008860 | *Hordeum vulgare* | chr6H:16102575-16104048 | 420 | 45.01 | 5.73 | 0 | Chloroplast | Inhibitory | - |
| HvSRP7-1 | HORVU7Hr1G008620 | *Hordeum vulgare* | chr7H:11238389-11239930 | 406 | 43.68 | 6.03 | 1 | Cytoplasm | Inhibitory | - |
| HvSRP7-2 | HORVU7Hr1G011420 | *Hordeum vulgare* | chr7H:16264762-16266027 | 181 | 20.03 | 5.89 | 0 | Chloroplast | Inhibitory | - |
| HvSRP7-3 | HORVU7Hr1G043530 | *Hordeum vulgare* | chr7H:132141003-132142590 | 432 | 46.40 | 6.53 | 0 | Cytoplasm | inhibitory | - |

+ indicates the presence of signal peptide; - indicates no signal peptide.
